# Phosphoproteome Profiling of Klebsiella pneumoniae under Zinc-Limited and Zinc-Replete Conditions

**DOI:** 10.1128/mra.00258-23

**Published:** 2023-06-26

**Authors:** Chelsea Reitzel, Arjun Sukumaran, Christina Zanetti, Benjamin Muselius, Jennifer Geddes-McAlister

**Affiliations:** a Department of Molecular and Cellular Biology, University of Guelph, Guelph, Ontario, Canada; University of Notre Dame

## Abstract

The bacterial pathogen Klebsiella pneumoniae causes nosocomial infections with the acquisition of multidrug resistance, impeding treatment options. This study investigated the effect of zinc limitation on the phosphoproteome of K. pneumoniae using quantitative mass spectrometry. New insight is provided into cellular signaling methods used by the pathogen to respond to nutrient-limited environments.

## ANNOUNCEMENT

Klebsiella pneumoniae is an opportunistic bacterial pathogen that colonizes the skin and gastrointestinal tract of healthy individuals without causing symptoms (1). However, immunocompromised individuals can develop pneumonia, septicemia, and urinary tract infections upon colonization by the bacterium (2). K. pneumoniae displays increasing rates of antibiotic resistance, resulting in limited treatment options and higher mortality for infected patients (3). As a result, novel antimicrobial strategies are needed.

Within bacteria, zinc is the most abundant nonredox transition metal, providing structural or catalytic functions for proteins (i.e., metalloproteins), with additional roles in metabolism, cell wall formation, and virulence (4–9). Our group previously reported the impact of zinc limitation on K. pneumoniae and revealed a connection with capsule formation and transcriptional regulation (4). Moreover, in Escherichia coli, zinc ion availability influences posttranslational modifications, such as phosphorylation, with downstream implications for various cellular responses (10). However, the implications of zinc levels on the phosphoproteome of K. pneumoniae have yet to be explored. Here, we applied mass spectrometry-based proteomics to profile the phosphoproteome of K. pneumoniae under zinc-limited and zinc-replete conditions and identify phosphorylation events. These data provide a better understanding of the mechanisms of cellular signaling and reveal potential antimicrobial targets for the disruption of important cellular regulation events.

**FIG 1:**
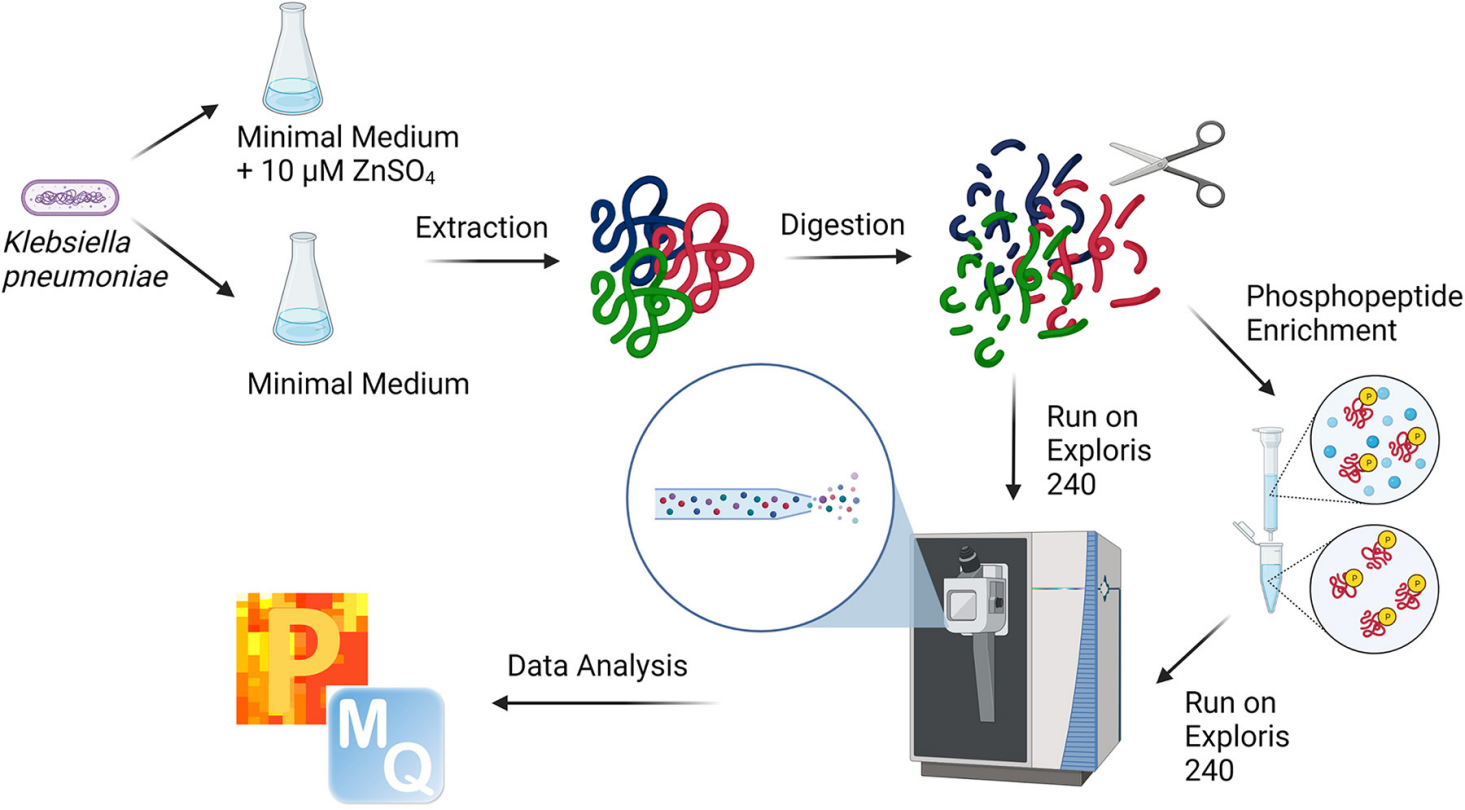
Workflow for total proteome and phosphoproteome profiling by bottom-up proteomics for K. pneumoniae under zinc-limited versus zinc-replete conditions. Figure generated using BioRender.

The proteome and phosphoproteome of K. pneumoniae (laboratory-adapted, ATCC 700721) were profiled under zinc-replete and zinc-limited conditions (Fig. 1). K. pneumoniae cells were grown in quadruplicate in 5 mL Luria-Bertani (LB) medium at 37°C with shaking (200 rpm) overnight. Cells were collected by centrifuging 0.5 mL culture at 3,500 × *g* and washing the precipitate twice with 0.5 mL M9 minimal medium (6.78 g/L Na_2_HPO_4_, 3 g/L KH_2_PO_4_, 0.5 g/L NaCl, 1 g/L NH_4_Cl, 0.4% [wt/vol] glucose, 2 mM MgSO_4_, 0.1 mM CaCl_2_, made to 1 L using Chelex 100-treated distilled water [dH_2_O] as previously described [11]). The cells were subcultured at a 1:100 ratio in 50 mL M9 medium or M9 medium supplemented with 10 μM zinc (ZnSO_4_) at 37°C with shaking (200 rpm). After 9 h (mid-log to early stationary phase), the cells were pelleted at 1,372 × *g*, and the pellet was washed twice with 5 mL phosphate-buffered saline (PBS). Total protein was extracted from the cell pellets as previously described (12). Briefly, the cell pellets were resuspended in 100 mM Tris-HCl (pH 8.5) proteinase inhibitor and PhosSTOP tablets, and sodium dodecyl sulfate (SDS; 2%). Probe sonication (30 s on/30 s off in an ice bath; 30% power) (Thermo Fisher Scientific) was used to lyse the cells, followed by the addition of dithiothreitol (DTT; 10 μM) (incubation at 95°C for 10 min with shaking at 800 rpm) for reduction and iodoacetamide (IAA; 55 mM) (incubation for 20 min in the dark at room temperature) for alkylation. Proteins were precipitated overnight (80% acetone at −20°C), centrifuged at 13,500 rpm for 10 min, washed twice with 0.5 mL 80% acetone, and air dried. The pellets were resuspended in 8 M urea/40 mM HEPES buffer for protein quantification (13), followed by trypsin/LysC protease mix digestion (1:50 enzyme/protein) overnight at room temperature. Next, the samples were subjected to phosphopeptide enrichment using TiO_2_ columns (Thermo Fisher Scientific; catalog number A32993) following the manufacturer’s instructions (approximately 800 μg for enrichment, 80 μg for total proteome), with peptide purification by STop And Go Extraction (STAGE) tips (formating) (StageTips) (14). Peptides (3 μg) were loaded onto Evosep tips according to the manufacturer’s instructions (15) and measured using a Thermo Scientific Orbitrap Exploris 240 mass spectrometer (15-cm PepSep column; precursor range, 400 to 2,000 *m/z* at 60,000 resolution; intensity threshold, 2.5e4; charge states, 2 to 8). Phosphopeptides were analyzed using a 44-min gradient and total proteome using an 88-min gradient.

MaxQuant v2.2.0.0 (16) was used to analyze the RAW files with default parameters (except as noted) using the Andromeda search engine (17) against K. pneumoniae subsp. *pneumoniae* serotype K52 protein sequences (5,126 sequences; 2 December 2022) from UniProt. Variable phosphorylation modification on S/T/Y/D/H amino acids was included with a neutral loss of H_3_O_4_P (mass 97.9768950 Da). Phosphopeptide abundance was normalized to the total proteome. Modified and unmodified peptides were included for protein quantification using label-free quantification (LFQ) (ratio count set to 1), with minimum peptide = 2 and match between runs (18) enabled. Perseus v2.0.7.0 (19) was used to analyze the output files. The data were filtered to remove potential contaminants, reverse peptides, and peptides only identified by site. Valid value filtering was used (peptides present in 3 of 4 replicates in least one condition). Only class I phosphopeptides were retained by filtering for localization probability greater than 75%. Statistical analysis was performed with Student’s *t* test (*P* < 0.05; false discovery rate, 0.05; S_0_ = 1). Using the total proteome data set, 1,869 proteins were identified (36% of the encoded proteome), and 24 phosphorylated proteins were measured (Table 1).

**TABLE 1:** Phosphorylated proteins detected in K. pneumoniae under zinc-limited versus zinc-replete conditions

Protein accession no.	Gene name	Multiplicity	Amino acid(s)	Position(s)	Description
A6T531	*pepD*	1	T	354	Aminoacyl-histidine dipeptidase
A6T596	*yhjT*	1	Y	412	Phospholipid/glycerol acyltransferase domain-containing protein
A6T5V4	KPN_00525	1	T	3	Aldo/keto reductase
A6T671	KPN_00642	1	S	144	Biotin sulfoxide reductase
A6T6D3	*pgm*	1	S	146	Phosphoglucomutase
A6T6Z6	*pflB*	1	T	442	Formate acetyltransferase
A6T726	*asnS*	1	D	404	Asparagine-tRNA ligase
A6T772	KPN_01007	2, 2	T, H	497, 498	Bacterial extracellular solute-binding protein
A6T789	KPN_01025	1	Y	30	Short-chain dehydrogenase/reductase
A6T7K6	*icdA*	1, 2	D, S	50, 113	Isocitrate dehydrogenase (NADP)
A6T818	KPN_01306	2, 2	T, T	331, 335	Transport protein
A6T870	KPN_01359	1	Y	44	Bacterial regulatory protein
A6T8U7	*ydfI*	1	T	465	Mannitol dehydrogenase
A6TA17	KPN_02010	1	Y	97	LysR family transcriptional regulator
A6TA64	KPN_02057	1	T	7	Short-chain dehydrogenase
A6TAG7	*ppsA*	1	T	419	Phosphoenolpyruvate synthase
A6TAQ3	KPN_02248	2, 2	T, H	48, 50	Type VI secretion system lipoprotein
A6TAU1	*yjiE*	2, 2	S, Y	19, 11	Transcriptional regulator (LysR family)
A6TAV6	KPN_02301	1	H	264	DUF403 domain-containing protein
A6TCE7	*hscA*	1	T	168	Chaperone protein
A6TD07	KPN_03077	1	T	25	Repressor of *galETK* operon
A6TEX3	*rpsJ*	1	T	44	30S ribosomal protein S10
A6TG01	*gyrB*	1	D	17	DNA gyrase subunit B
A6TH76	*yjeP*	1	D	406	Periplasmic binding protein

### Data availability.

The RAW and affiliated files are publicly available through the PRIDE partner database for the ProteomeXchange consortium (accession number PXD041015; http://www.ebi.ac.uk/pride/archive/projects/PXD041015).
